# The CHO Cell Clustering Response to Pertussis Toxin: History of Its Discovery and Recent Developments in Its Use

**DOI:** 10.3390/toxins13110815

**Published:** 2021-11-19

**Authors:** Mary C. Gray, Richard L. Guerrant, Erik L. Hewlett

**Affiliations:** Division of Infectious Diseases and International Health, School of Medicine, University of Virginia, Charlottesville, VA 22904, USA; mrc6r@virginia.edu (M.C.G.); rlg9a@virginia.edu (R.L.G.)

**Keywords:** CHO cells, cluster response, impedance, pertussis toxin

## Abstract

Chinese hamster ovary (CHO) cells respond to pertussis toxin (PT) with a novel clustering pattern, which is dependent on biologically active PT. Since its description in 1983, this cellular response has been refined and used extensively for detection and quantification of PT activity, as well as anti-PT antibodies. There are limitations, however, in the use of this phenomenon as originally described. They are: (1) a subjective, observer-dependent scoring system; (2) the requirement for 16–24 h incubation in order for the response to be clearly detectable; and (3) apparent interference from non-toxin materials. To overcome these limitations, a number of alternative in vitro assays for PT, using CHO cells or other cell types, have been developed and are described elsewhere in this publication. In addressing the challenges associated with the CHO cell assay, we discovered that changes in the electrical impedance-based “normalized cell index” of PT-treated CHO cells obtained with the ACEA xCELLigence instrument enable objective detection/quantification of the PT-induced effect in as little as 3–4 h. To the best of our knowledge, the molecular basis for this intriguing response remains unknown. We present here electron microscopic (EM) images of control and PT-treated cells, which suggest some potential molecular mechanisms.

## 1. Introduction

The morphologic response of Chinese hamster ovary (CHO) cells to pertussis toxin (PT) has been used extensively for quantification of PT and anti-PT antibodies. This novel phenomenon was discovered serendipitously in the early 1980s as part of an early exploration of the mechanism of PT action [1]. In 1980, Erik Hewlett joined the Division of Geographic Medicine in the Department of Medicine, University of Virginia School of Medicine. This newly formed unit was headed by Dr. Richard Guerrant, an established investigator in the field of diarrheal diseases, especially those caused by cholera toxin (CT) and *E. coli* heat-labile enterotoxin (LT) [2,3,4,5,6]. Geographic Medicine at the University of Virginia was one of 14 Divisions of Geographic Medicine that were created and funded, around the world, as part of the Rockefeller Foundation “Great Neglected Diseases Network”, led by Dr. Kenneth Warren [7]. Soon after the first publication describing the effect of PT on CHO cells, Mary Gray joined the Hewlett Lab, where she contributed scientifically and served as advisor and mentor to students and fellows, in addition to managing the lab for more than 30 years. She was central to the refinement and use of this assay for research and quality-control purposes.

**Figure 1:**
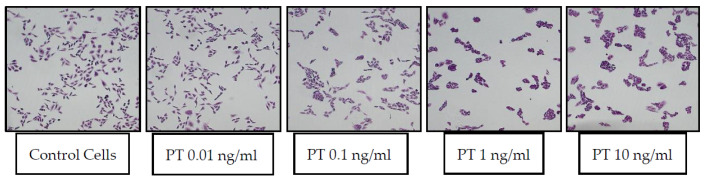
PT-treated CHO cells appear in clusters, while control cells remain spatially dispersed. CHO cells were plated and cultured overnight, then buffer or PT was added. After 24 h of incubation, the medium was removed and the cells were fixed and treated with 2% Giemsa stain for 20 min. Images were obtained by light microscopy.

Following the discovery by Hsie and Puck [8] that CHO cells respond to increased cyclic AMP with an elongated morphology (stretching response), Guerrant et al. used that morphologic change for detection of CT and LT and antibodies against them [9,10]. Since the CHO-stretching assay was being used in the Guerrant lab in the late 1970s and early 1980s, it seemed logical to begin a study of PT effects, which were thought at the time to involve, at least in part, changes in cAMP levels, by evaluating them in the existing CHO cell assay. The response of CHO cells to PT was examined, using CT as a positive control. Unexpectedly, after 24 h, there was no “stretching” of the PT-treated CHO cells; rather, they appeared in clusters, with the morphology of individual cells (at the level of light microscopy) not appearing grossly different from control cells [1]. Representative images of control CHO cells and those treated with a range of PT concentrations (0.01–10 ng/mL) are shown in Figure 1. 

**Figure 2:**
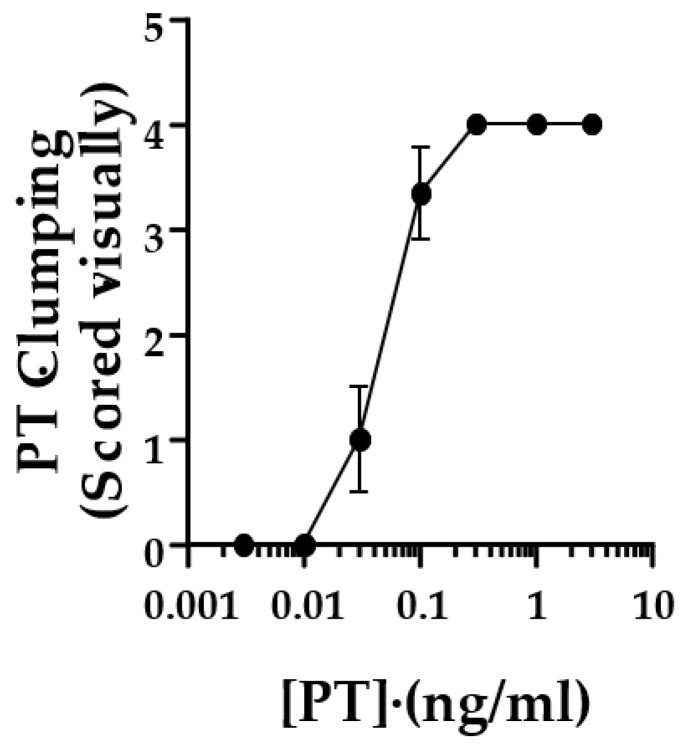
Scoring of the clustering/clumping response of CHO cells to PT. CHO cells at 2.5 × 10^4^/mL were grown overnight at 37 °C, 5% CO_2_. PT was added at indicated concentration to duplicate wells and incubated for 24 h at 37 °C, 5% CO_2_. Cells were stained with Giemsa stain and read visually for clustering. A score of 2 is given if the majority of cells appearing in clusters, 1 for >30% of cells in clusters, 0 for <30% of cells in clusters. Scores of duplicate wells were added together, yielding a score of 0–4. A score of 3 is considered positive. Data are from three independent experiments.

To quantify the responses in the original studies, control and test wells were scored by two observers, with a positive cluster effect scored as “2”, equivocal as “1” and no response as “0”. Scores from the two observers were added together, yielding a potential range of scores, 0–4. This was later modified to eliminate the requirement for more than one observer. Each well was scored as described in Methods, and values from duplicate wells were combined to yield the same range of 0–4. A combined score of 3 or greater was considered positive. Figure 2 shows the scoring of this assay with the lot of List Laboratories PT used for the images in Figure 1.

In the original studies, as anticipated, CHO cells exhibited the previously described stretching response to as little as 0.6 ng/mL CT. Cells treated with CT and PT were both clustered and stretched, indicating, importantly, that the two responses are separate and distinct and not significantly affected by the occurrence of the other [1]. The absence of stretching in PT-treated cells underscored the fact that PT is not acting through increased intracellular cAMP levels (discussed in greater detail below) and the absence of cluster response in CT-treated cells makes it unlikely that this phenomenon is controlled by cAMP. Importantly, in studies carried out subsequently, CHO cells were treated with either adenylate cyclase toxin (ACT) from *B. pertussis* to increase cAMP levels or PT or both. As with CT treatment, the CHO cells exposed to ACT demonstrated the stretching response only and those exposed to ACT and PT were both clustered and stretched (data not shown), as was observed with CT and PT in Hewlett et al. [1]. The lack of relationship between cAMP levels and the cluster response was confirmed subsequently by Zamith et al. [11].

A time course of the cellular response (presented in original studies, [1]) revealed that the PT-clustering effect could be detected at 16+ h, even after exposure of cells to PT for as little as 10 min (followed by washing and fresh medium), and a PT concentration as low as 120 pg/mL (the sensitivity has subsequently been established to be ≥100 pg/mL). PT did not appear to be acting as an agglutinogen, in that addition of toxin to CHO cells, in a single-cell suspension or plated, had no immediate effect. This was in contrast to the effect of concanavalin A, which was used as a positive control and elicited rapid agglutination of CHO cells in suspension. There did, however, appear to be an effect on the surface properties of PT-treated CHO cells as the toxin effects were developing. After exposure to PT (70 ng/mL for 20 h) and development of the cluster response, CHO cells were dispersed to a single-cell suspension and, in contrast to control cells, displayed spontaneous aggregation. Based on these observations and the novelty of the cell pattern, the clustering response was proposed as an in vitro assay for quantification of PT and detection of anti-PT antibodies [1].

Soon after the original publication describing the CHO cell response to PT, Gillenius et al., a group experienced in assay development, described a refined version of the CHO cell assay for the purposes of detecting/quantifying PT and identifying PT-neutralizing activity in serum from convalescent patients or immunized individuals [12]. In 1987, Burns et al. showed that this effect is dependent on the presence of the A subunit of PT and its ADP ribosyl transferase activity [13]. In 1989, Fujiwara and Iwasa described further refinements in the assay and statistical analysis of data obtained therefrom [14]. 

During its use for these purposes over the past 35+ years, the CHO cell assay has been evaluated and assessed for its appropriateness in detecting and quantifying PT in a number of settings, most recently as residual activity in acellular pertussis vaccines (as illustrated in references [11,15,16,17,18,19,20,21,22]. This “semi-quantitative assay” is limited specifically by the subjective scoring system required for characterizing a positive response and by the requirement of 16+ hours for the phenomenon to develop in order to be identifiable by light microscopy. A review of the limitations to using this response as an assay and the subsequent development and testing of alternative approaches, including objective, observer-independent scoring systems, are presented by Hoonakker [15] and elsewhere in this issue). 

To address ongoing concerns about the CHO cell assay, we took a different approach, using the ACEA xCELLigence to detect and quantify the changes in impedance of CHO cells elicited by PT. In addition, we continue to be intrigued by the mechanism through which this phenomenon occurs and provide herein EM images of control and PT-treated CHO cells to prompt thought and discussion on this topic.

## 2. Results and Discussion

**Figure 3:**
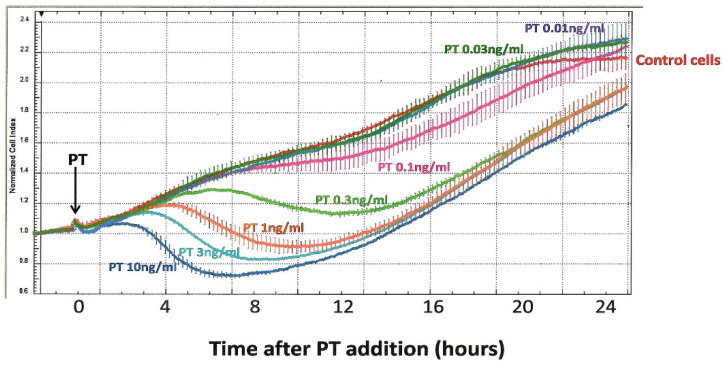
Effect of PT on normalized cell index (impedance) of CHO cells, as measured with the ACEA xCELLigence System. CHO cells were plated at 5 × 10^4^/mL in an E-plate 16 and monitored for 25 h. PT was added (indicated by the arrow), at specified concentrations, and impedance readings were taken every 3 min; each concentration of PT was assayed in duplicate.

Concerns about the above-listed limitations in the CHO cell clustering assay led us, more than a decade ago, to pursue alternative approaches for timely and objective detection of the CHO cell response to PT. Through the UVA School of Medicine Biomolecular Analysis Facility, which provides specialized, expensive equipment in a central facility, for use by individual investigators, we were able to employ an ACEA xCELLigence instrument. This novel technology uses special multi-well, cell-culture plates (E-Plates) with embedded gold electrodes to monitor impedance produced by a population of plated cells. Since changes in cell number or morphology/contact with the well surface can be detected, we hypothesized that this approach could be useful to monitor the effects of PT on CHO cells. Indeed, as shown in Figure 3, the xCELLigence technology provides a new perspective on the CHO cell response. In these experiments, PT, at final concentrations of 0.01–10 ng/mL, was added to CHO cells, which had been plated and incubated overnight; measurements were begun at the time of toxin addition.

Using this protocol, control CHO cells demonstrate a progressive increase in impedance (“normalized cell index”), which continues for more than 20 h of observation, as the cell number increases, and then begins to plateau. When PT is added to these cells, it elicits a striking, concentration-dependent decrease in normalized cell index, with the effect of the highest tested concentration of PT (10 ng/mL) apparent in 3–4 h. 

**Figure 4:**
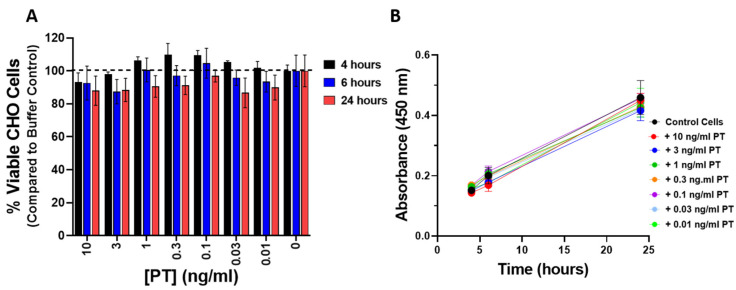
(**A**) PT treatment does not affect the viability of CHO cells. CHO cells were plated in 96-well tissue culture plate at 2.5 × 10^4^/mL and grown overnight. PT was added and incubated for indicated time at 37 °C, 5% CO_2_. Percent viable cells was determined using Cell Counting Kit-8 (CCK-8, Dojindo Molecular Technologies). Data represent three replicates at each concentration and time and is representative of two similar experiments. (**B**) PT treatment does not affect the rate of proliferation of CHO cells over the course of 24 h. CHO cells were plated in 96-well tissue culture plate at 2.5 × 10^4^/mL and grown overnight. PT was added and cells incubated for indicated time at 37 °C, 5% CO_2_. CCK-8 solution (Dojindo Molecular Technologies was added and absorbance read at 450 nm. An increase in absorbance indicates an increase in cell number. Data represent three replicates of each concentration and time and is representative of two similar experiments.

To be sure that the observed changes in impedance were not a result of cytotoxicity or a change in the rate of proliferation elicited by PT, CHO cells were treated with PT (0.01–10 ng/mL) for 4, 6, and 24 h and compared to control cells using the CCK-8 cell viability assay. As shown in Figure 4A, PT treatment of CHO cells did not affect the number of viable cells. In addition, there was no effect on the rate of proliferation in cells treated with PT, compared to control cells, over the course of 24 h (Figure 4B). 

**Figure 5:**
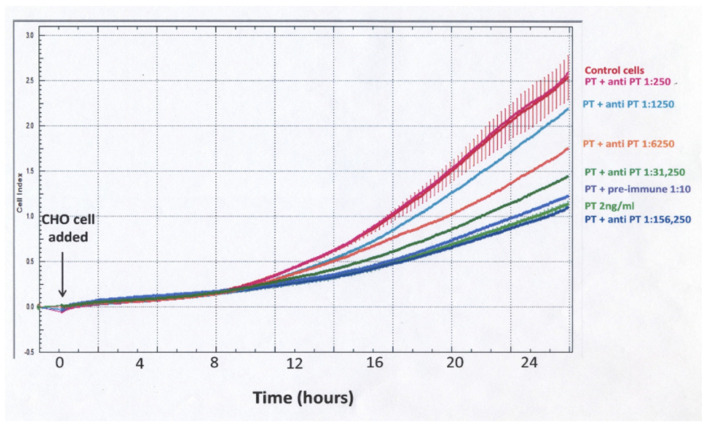
Characterization of the effect of rabbit anti-PT serum on the CHO cell response to PT using cell impedance. Polyclonal anti-PT antibody, at indicated dilutions, was incubated with PT (2 ng/mL) in the wells of an E-plate 16 for 3 h at 37 °C. CHO cells at 5 × 10^4^/mL were then added and impedance measured every 3 min for 24 h using the ACEA xCELLigence system.

When using cell impedance to test for neutralization of the PT effect by rabbit anti-PT serum, a different experimental protocol was used (Figure 5). The PT–antibody mixture was incubated for 3 h in the designated wells and then CHO cells were added and cell impedance, as reflected in normalized cell index, was followed for 24 h. With this protocol, there was a gradual increase for control and PT-treated cells, but, unlike the protocol demonstrated in Figure 3, the differences between control and PT-treated cells were much less striking and not apparent until 10–12 h. These data demonstrate that sensitivity, objectivity and rapidity make impedance measurements of plated, growing CHO cells a strong candidate for obtaining objective detection and quantification of PT biological activity and anti-toxin antibodies. An impedance endpoint can be defined and agreed upon among users and the frequent measurements during the period of observation provide much more information than a single endpoint value.

This work was presented at the “International Workshop on Alternatives to the Murine Histamine Sensitization Test for Acellular Pertussis Vaccines: State of the Science and the Path Forward”, held at the National Institutes of Health, Bethesda, MD, USA, in November 2012 [22] and at the “Real-Time, Cell-based Assay System Workshop” during the annual meeting of The American Society of Cancer Research in Washington DC, USA in 2013. Importantly, Bernardo et al. [23] recently published a comprehensive and compelling description of use of the “xCELLigence real time cell analysis” for detection of PT-induced clustering in CHO cells. The data presented in that publication employed a protocol similar to that used in Figure 5, so that opacity of PT in medium + antibodies could be used to determine instrument background, prior to addition of CHO cells. In comparing the two approaches, we found that addition of PT to plated and growing CHO cells, as shown in Figure 3 above, is at least as sensitive and reveals the PT-induced changes more rapidly (3–4 h, depending on PT concentration) than plating cells in the presence of PT. Nevertheless, the data presented in Bernardo et al. [23] and herein make a strong case for xCELLigence technology serving as the missing link for making the CHO cell assay rapid, reliable and no longer dependent on subjective scoring. 

An important aspect of the CHO cell response is that its molecular mechanism/s remain unknown. PT affects target cells by ADP-ribosylation of several G-proteins, thereby impairing their ability to function in coupling surface receptors to effector mechanisms. Xu and Barbieri showed that chloroquine, monensin and nocodazole, but not cytochalasin-D, inhibits PT-mediated ADP-ribosylation of Gi-2 and Gi-3 [24]. Although prevention of ADP-ribosylation would be expected to prevent the cluster response, this study did not address the process downstream from the ADP-ribosylation step [24]. 

Zamith et al. noted that treatment with PT for 15 min or 48 h did not alter cAMP levels, but they raised the possibility that there might be effects of PT on CHO cell cGMP levels that could be responsible for or contributing to the morphologic effects [11]. While the involvement of cGMP has not been addressed directly, it is not likely in light of the data from Crane et al. [25] in examining the effect of PT on the activation of guanylate cyclase by *E. coli* heat-stable toxin (STa). They found no effect of PT on either unstimulated (basal) or STa-elicited cGMP levels in T84 cells. 

**Figure 6:**
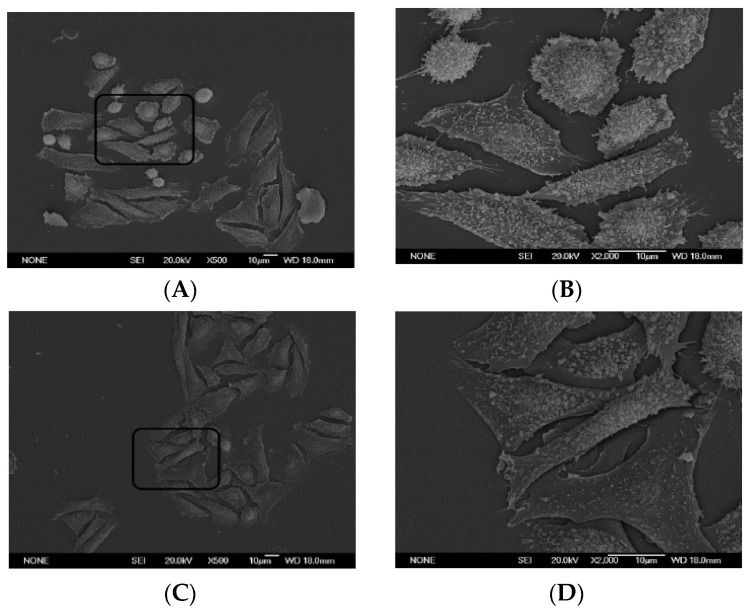
Electron microscopy reveals that unlike control cells, PT-treated CHO cells appear to overlap neighboring cells. CHO cells were plated on glass coverslips at 5 × 10^4^/mL and allowed to grow for 24 h. PT (10 ng/mL) was added and the mixture incubated for 24 h. Cells were fixed and images obtained at ×500 and ×2000 with scanning electron microscopy. (**A**) Control CHO cells (500×); (**B**) Control CHO cells (2000×); (**C**) PT-treated CHO cells 500×; (**D**) PT-treated CHO cells (2000×).

To our knowledge, there is not, at present, a recognized link between known G-protein functions and the morphologic response elicited by PT in these cells. For that reason, we continued exploration of the PT effect by examining control and PT-treated CHO cells by electron microscopy (Figure 6) to determine if the ultrastructure of the responding cells provides any clues. Figure 6A,B show control CHO cells at 500× and 2000× magnification, respectively. It is of note that although control cells exhibit occasional surface appendages that are touching other cells, there is extensive visible space between the cells (Figure 6B). In contrast, the PT-treated cells, which are exhibiting the characteristic cluster pattern, are not well separated, with some cells appearing to be overlapping/on top of one another (Figure 6C,D). While these images can only suggest additional approaches to identifying the molecular mechanism by which PT elicits this striking effect, they do raise interesting possibilities. Normal cells move apart after cell division, in order not to impair further proliferation by the process known as “contact inhibition” [26]. The PT-treated cells appear either to be unable to move apart or to have lost the need to do so without limiting their proliferative ability. That these cells are not dying or being limited in their proliferative capacity is illustrated in Figure 4. The numbers of viable CHO cells and their growth rates are not significantly affected. Together, these data/images provide an additional perspective on the PT effect, which includes notable differences in cell surface morphology, as well as intercellular spatial relationships, but does not appear to involve alterations in cell proliferation or cell death. 

It has been stated frequently that “PT increases cAMP levels in target cells”, with the implication that this is or is one of its principal mechanisms of action. This incorrect concept has resulted in significant confusion in the field. Hazeki and Ui [27] showed in 1981 that although PT can enhance an agonist-elicited cAMP response, it does not affect basal/resting cAMP in treated cells. Hoonakker et al. employed this effect when developing the cAMP assay in which PT produces a significant enhancement of the response of A10 cells to isoprenaline or forskolin, but also does not affect basal cAMP [16]. Similarly, Zamith et al. found no concentration of cholera toxin, forskolin or dibutyryl cAMP that elicited the clustering response [11]. Furthermore, data from these and numerous other papers are consistent with the point that, in contrast to CT, PT does not elicit CHO stretching, which would be observed if cAMP levels were significantly affected. Only when there is tonic inhibition of AC by endogenous or exogenous molecules does PT affect cAMP levels, by blocking that inhibitory input. This point is further illustrated in Olansky et al. [28] showing that PT-induced lipolysis in rat adipocytes results from blocking the inhibitory effect of endogenously produced adenosine and does not occur when adenosine is eliminated by addition of adenosine deaminase. Therefore, at present state of knowledge, it is not appropriate to describe PT as increasing cAMP levels, as its primary mechanism of action or at all, without consideration of the other variables in play.

In summary, the effect of PT on CHO cells in this assay has served as the basis for detecting/measuring PT activity for almost forty years, but its use for some applications has been limited by the subjective scoring system and by the duration of time required for the response to be detectible and measurable. Use of cell impedance for this purpose provides an objective approach and a reduction in the time required, as well as much greater amount of data than a single endpoint reading, thereby increasing the likelihood that this phenomenon can be used appropriately for these purposes.

## 3. Materials and Methods

CHO Cell Cluster Response—Chinese Hamster Ovary (CHO) K-1 cells were obtained from ATCC and maintained in F12 + 10% FBS. For CHO cell-PT assays, CHO cells were plated in a 96-well tissue culture plate at 2.5 × 10^4^/mL in F12 + 1% heat-inactivated (HI) FBS and grown overnight at 37 °C, 5% CO_2_. PT preparations purified in the Hewlett lab and obtained from List Laboratories were comparable in activity. PT was added at indicated concentrations to duplicate wells and the mixture incubated for 24 h at 37 °C, 5% CO_2_. Cells were stained with 2% Giemsa Stain and read visually by light microscopy for the cluster response. A score of 2 is given if the majority of the cells appear clustered; a score of 1, if >30% of the cells are clustered; and a score of 0, if <30% of cells are clustered. Scores of duplicate wells are added together, yielding a range of 0–4, with a score of 3 considered positive.

Impedance Measurements—Real-Time Cell Analysis (RTCA) measurements of impedance were made using the ACEA xCELLigence System, which employs noninvasive gold microelectrodes in a multi-well plate format, providing a sensitive readout of cell number, size/morphology, and attachment. For experiments examining PT-effects on adherent CHO cells, cells were plated in an E-Plate 16 (Aligent, Santa Clara, CA, USA) at 5 × 10^4^/mL in F12 + 1% HI FBS and monitored for 24 h. PT, at indicated concentrations, was added and readings were taken every 3 min over the course of 24 h. For experiments examining the effects of anti-PT antibodies, polyclonal, anti-PT antibody was incubated with PT (2 ng/mL) in the wells of an E-Plate for 3 h at 37 °C. CHO cells at 5 × 10^4^/mL were then added and impedance measured every 3 min for 24 h.

CHO Cell Viability and Proliferation—Viability and proliferation of CHO cells during the time intervals of these experiments were measured using Cell Counting Kit-8 (CCK8) from Dojindo. CCK8 is a sensitive colorimetric assay which allows accurate live cell counting in cell proliferation or cytotoxicity assay applications. CHO cells were plated and treated with PT as indicated. CCK8 was added at indicated times, incubated for 1 h at 37 °C, and absorbance at 450 nm measured.

Electron Microscopy—CHO cells were plated on ethanol-cleaned glass coverslips at 5 × 10^4^/mL and allowed to grow for 24 h. PT (10 ng/mL) was added and incubated for 24 h. Cells were fixed and images obtained at ×500 and ×2000 with scanning electron microscopy. 

## 4. Conclusions

Limitations to using the cluster response of CHO cells to PT, as originally described, can be overcome by the detection of changes in electrical impedance of the CHO cells as a function of time, following PT addition. This objective measurement technique appears to enable detection of the PT-elicited changes in a much shorter time frame.

## Data Availability

Not applicable.

## Addendum

We have recently become aware of additional publications [29,30,31,32] that appear to be very relevant to the work described herein. In 1992, Verschueren et al. [32] described a “scatter factor”, which, when partially purified from CHO cell supernate, caused both CHO and MDCK cells to move apart and more importantly, counteracted the cluster response of CHO cells to PT. No follow-on information was found and thus the potential relationship of this “factor” to the CHO response elicited by PT remains unknown. In a manuscript published very recently in Toxins (29), Overgaard et al. described the effects of ArtAB toxin from *Salmonella* Typhimurium and the isolated “B” component of that toxin. The purified holotoxin (ArtAB), which has homology with other ADP-ribosylating toxins, such as PT and CT, caused a clustering response in CHO cells very similar to that produced by PT. Furthermore, that same molecule also caused elongation of Vero cells, very similar to that produced by CT effect on CHO cells. Paramonov et al. described an elegant construct for detection of PT activity [31]. Using HEK293 cells engineered to co-express an inhibitory G-protein-coupled receptor (somatostatin receptor 2), which is controllable by an agonist, octreotide, and a luminescent cAMP probe. In these cells, activation of adenylate cyclase by forskolin in the presence of octreotide results in a reduced signal relative to forskolin alone, due to the inhibitory effect of octreotide. After exposure to PT, however, the response to forskolin is enhanced and the inhibitory effect of octreotide is lost. Importantly, the studies of Paramonov et al. also found that PT alone does not increase cAMP in these proliferating, engineered HEK293 cells [31]. Most recently, Hoonaker et al. employed “nearest neighbor” image analysis to provide an observer-independent method for reading and quantifying the CHO cell response to PT [29]. Although these fascinating publications do not answer the questions raised herein on the mechanism of the PT effect, they certainly do raise additional questions and, hopefully, provide new tools and ways to think about the cluster phenomenon produced by PT.
